# Recent developments in the use of γ -H2AX as a quantitative DNA double-strand break biomarker

**DOI:** 10.18632/aging.100284

**Published:** 2011-02-11

**Authors:** Christophe E. Redon, Asako J. Nakamura, Olga A. Martin, Palak R. Parekh, Urbain S. Weyemi, William M. Bonner

**Affiliations:** Laboratory of Molecular Pharmacology, Center for Cancer Research, National Cancer Institute, Bethesda, MD 20892, USA

**Keywords:** DNA damage, DNA repair, γ -H2AX, double-strand break, biomarker, cancer, senescent cells

## Abstract

The past year has seen considerable developments in the use of the DNA double-strand breaks (DSBs) to evaluate genome alterations in cells undergoing a variety of genotoxic stresses in vitro and in vivo. When the γ -H2AX foci which mark the DSBs are stained, individual breaks are detectible, making the assay suitable for situations requiring great sensitivity. While the methods for the detection of γ -H2AX foci are still evolving, particularly for in vivo detection, the basic assay has proven to be useful in several diverse areas of research. We will highlight recent developments of the assay in four areas: radiation biodosimetry, the evaluation or validation of new cancer drugs in clinical studies, chronic inflammation, and environmental genotoxicity.

## Background

The creation of a double-strand break (DSB) in eukaryotic cells is generally accompanied by the formation of hundreds of histone γ-H2AX (H2AX-S139PO4 in humans) molecules in the chromatin flanking the DSB site [1]. Antibodies to γ-H2AX allow the visualization of a “focus” at the DSB site. The foci also serve as sites for accumulation of other proteins involved in DSB repair, leading to the suggestion that the foci have roles in signal amplification and the accumulation of DNA repair factors that, in turn, facilitate chromatin remodeling, cell cycle checkpoint functioning, sister chromatid-dependent recombin-ational repair and chromatin anchoring to prevent the dissociation of broken ends [reviewed in [1-5]]. However, although γ-H2AX appears to be a principal player in the DNA damage response and necessary for the initial rapid phase of DSB repair, mice lacking H2AX, while hypersensitive to ionizing radiation, are still capable of DNA damage signaling and repair [6]. The viability of the H2AX-null mouse indicates that H2AX is not essential for homologous recombination or non-homologous end joining itself. However, these mice suffer from two major deficits—a lack of class switch recombination during immune system development and a lack of sperm production in males. The former process is known to involve DSBs and genomic rearrangements. In the testes of H2AX-null males, autosome pairing and synapsis appear to take place normally, but the X and Y chromosomes fail to form a condensed sex body [1,6].

**Figure 1.**
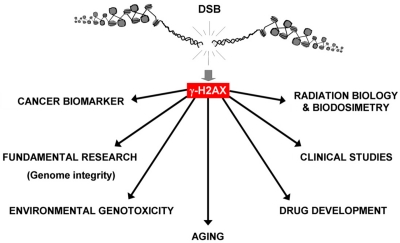
Applications for γ-H2AX detection. Because of its sensitivity, the γ-H2AX assay is now utilized in many research areas “from benchtop to bedside” by researchers and clinicians. In addition to being widely used for fundamental research (study of genome stability, DNA repair, etc.) in the last decade, γ-H2AX was identified as a biomarker for cancer (and premalignant lesions) and used to better understand aging. Additionally, γ-H2AX has been developed for radiation biology and biodosimetry for drug development and clinical studies (chemotherapy, the impact of chronic inflammation and diabetes on genome integrity). Finally, γ-H2AX measurement is an efficient and sensitive genotoxic assay for environmental studies.

Potential practical uses of γ-H2AX foci formation in cells and tissues have been apparent from soon after its discovery. The amplified response makes it possible to easily visualize individual DSBs in cell nuclei, making γ-H2AX foci staining more sensitive than other methods of DSB detection [7]. The realization that DSBs, whether alone or as one type of a spectrum of DNA lesions, are involved in many processes that disturb cellular homeostasis has led to broadening use of γ-H2AX foci detection beyond basic research. It has been used as a biomarker for aging and cancer, and a biodosimeter for drug development, radiation exposure and for clinical trials for cancer chemo- and radiotherapy. Finally, other emerging uses for γ-H2AX include detection of toxic environmental agents and the detection of chronic inflammation (Figure 1). In this report we highlight recent advances in four areas utilizing γ-H2AX detection.

### Radiation biodosimetry

Since ionizing radiation induces DSBs among a broad spectrum of DNA lesions, assessment of the biologic response to radiation exposure is a straightforward application in the use of γ-H2AX as a biodosimeter. Detection of radiation-induced DSBs *in vivo* using the γ-H2AX assay has been utilized as a tool for dose estimation in the clinic for localized irradiation with both high (radiotherapy) and low doses (X-ray examination, computed tomography (CT) scan, etc) [see [7-9] as examples]. γ-H2AX may be used to improve the conditions for patients undergoing radiation treatments, for example as a tool to estimate individual radiosensitivity, scattering or abscopal effects in normal tissues (discussed below). Kuefner *et al*. (2010) followed γ-H2AX foci formation during different cardiac CT protocols and showed that it is important to carefully adapt these protocols to avoid unnecessary X-ray-induced DSBs [7,10]. While the γ-H2AX assay gave results which supported the exposures estimated by physical modeling, it takes into account various biological factors not available in physical modeling, giving more confidence to these measurements.

However, while studies reported the use of γ -H2AX foci induction following exposure to therapeutic doses of ionizing radiation [9,11-15], how the assay would perform at higher doses, particularly in humans, remained unclear. Recently, the opportunity arose to evaluate γ -H2AX biodosimetry in a study using non-human primates subjected to total-body irradiation in the non-lethal to lethal dose ranges [16]. Using realistic scenarios for accidental exposures, the authors showed that γ -H2AX analysis in lymphocytes and plucked hair follicles (eyebrows and whiskers) may be useful for estimation of radiation dose at times at least 4-days post-exposure at doses of 3.5 Gy and above. In addition, the Center for High-Throughput Minimally Invasive Radiation Biodosimetry has developed a fully automated high-throughput system, the RABIT (Rapid Automated Biodosimetry Tool) to respond to major radiological accidents. The RABIT is able to perform the γ -H2AX analysis for radiation biodosimetry of up to 30,000 blood samples a day and is intended to fully automate the γ -H2AX assay, from the isolation of human blood lymphocytes to the immunolabeling of γ -H2AX and image acquisition [17,18].

### Drug biodosimeter

While ionizing radiation and a few cancer drugs create DSBs directly in the DNA, many drugs induce DSB formation indirectly through interference with DNA replication and transcription [1,19,20]. A well characterized example involves the drug camptothecin that traps topoisomerase I (top1) in DNA complexes which in replicating cells often result in DSB formation as replication forks collide with the trapped top1 complexes [1,21].

While chemotherapeutic agents are given with a particular type of tumor in mind, every cell in the body may be exposed to the agent. Measuring the amount of DNA damage in a patient's tissues soon after drug administration would allow researchers and clinicians to determine the efficacy of a drug to create DNA damage and genomic instability in the cells of a particular patient. Such information could help “personalize” the doses and delivery of a single drug or combinations of drugs to individual patients in terms of DNA damage efficiencies, and further correlate of these results with data on tumor regression and ultimate patient survival. This may result in optimized protocols that improve patient outcomes.

The practicality of γ-H2AX as a reproducible pharmacodynamic marker of top 1 inhibitor activity has been evaluated with an assay developed and validated in two laboratories [22]. Using three structurally related indenoisoquinoline Top1 inhibitors in human xenograft mouse models, the assay gave significant responses in tumor biopsies and in skin snips at the mouse equivalents of clinically relevant doses. One advantage of this assay is that results on drug activities were obtained four hours after administration, more quickly than waiting for visible tumor responses. On the basis of this assay, two of the three compounds were selected for further clinical evaluation.

However, the specificity of many cancer drugs for replicating cells creates a problem concerning appropriate tissues to sample for measuring γ-H2AX foci formation. Sampling the tumor may be the most direct means to measure the efficiency of a cancer therapy, but collecting tumor biopsies is often risky and invasive for the patient. However, tumor cells are shed by many tumors into the peripheral blood of the patients. Monitoring γ -H2AX levels in a patient's circulating tumor cells (CTCs) following cancer treatment has been evaluated and may be a promising technique for following the pharmacodynamic effects of anticancer therapies [23].

The lymphocytes present in peripheral blood have been utilized to detect γ -H2AX formation during cancer treatment, but these terminally differentiated cells may respond poorly to anticancer drugs that interfere with DNA metabolism. However, recent work has shown that plucked hair bulbs, which contain replicating cells and can be obtained non-invasively, may be utilized to monitor DSB formation *in vivo* after drug administration [24,25]. The indenoquinoline study showed that hair follicles in skin snips, used instead of plucked hair bulbs in athymic nude mice, responded similarly to the tumor, suggesting that plucked hair bulbs may be an appropriate surrogate tissue [22]. However, while both the tumor and hair bulb contain replicating cells, those of the tumor may have genetic alterations that make their responses to a particular agent different than that of normal replicating cells, both in terms of γ-H2AX foci formation and cell survival. This is a question requiring further research—how does the response of a surrogate tissue correlate with the response of the tumor and the patient and can it be predictive of that response.

### Distant DNA damage and chronic inflammation

The high sensitivity of the γ-H2AX foci assay has enabled researchers to measure low levels of DNA damage. Intercellular communication among cells in a culture or organism, where some of the cells have been damaged, has been found to result in the presence of low levels of DNA damage in cells peripheral to the damaged cells. For example, the radiation-induced bystander effect refers to the situation where a larger fraction of the cell population dies compared to the fraction hit by ionizing particles [26]. The effect can be demonstrated when a few cells in a culture are targeted with alpha particles, when an irradiated culture is mixed with an unirradiated one, and when the media conditioned on an irradiated culture is transferred to an unirradiated culture [27]. This last method indicates that substances released into the media from the irradiated culture are inducing the effects in the recipient normal, bystander culture. Bystander cells have been shown to exhibit greater numbers of chromosomal aberrations, micronuclei and γ-H2AX foci as well as increased mortality [28]. While the incidence of these defects is often just a few-fold elevated over the control values, the increase can be measured with γ-H2AX foci.

Cells, not exposed to genotoxic agents, but which are aberrant in some way may also induce a bystander effect in normal cells. γ-H2AX levels are elevated in cancer and aging cells in which it marks both DSBs and abnormal telomeres [29]. Media conditioned on cultures of both aging and tumor cells were found to induce elevated levels of γ-H2AX foci in normal cells. These results suggest that cells may constantly be releasing factors into their surroundings which affect other cells, at least in culture [30].

Recently, a tumor-induced bystander effect has been demonstrated in mice [31]. Syngenetic tumors were implanted subcutaneously in normal mice, and after two weeks, various organs of the mice were analyzed for DNA DSBs and another type of serious DNA lesions, oxidative clustered DNA lesions (OCDLs). Elevated levels of γ-H2AX foci were found in tissues of the gastrointestinal tract and skin including hair follicles; elevated levels of OCDLs were more widely distributed, present in lung and ovary as well as the gastrointestinal tissues and skin. A possible explanation for these results is that DSB formation is more common in proliferating cells where replication forks may encounter single strand breaks with a resulting DSB, while OCDL formation is less dependent on DNA metabolism. Gastrointestinal tract tissues and hair follicles contain higher fractions of proliferating cells compared to other tissues like ovary and lung.

In contrast to cell culture, this distant bystander effect in mice was found to be dependent on the immune system. Serum taken from tumor-bearing mice exhibited elevated levels of several chemokines involved in macrophage activation, and F4/80+ macrophages were found in the affected distant tissues of tumor-bearing mice compared to their control cohorts. Furthermore, when this experiment was repeated in mice lacking the gene for one of these chemokines, monocyte chemo-tactic protein 1 (MCP-1/CCL2), levels of DNA damage in distant tissues were not elevated. These results strengthen a connection between chronic inflammation and the presence of elevated levels of DNA damage induced by the production/release in tissues of oxidative molecules (i.e. by activated macrophages).

While these results were obtained in a mouse model system, similar processes appear to be occurring in humans. There is a relationship between chronic inflammation and obesity [32], characterized by increased secretion of proinflammatory cytokines such as interleukin-(IL)-6, IL-8 and MCP-1/CCL2, abundant infiltration of activated macrophages into adipose tissue and obesity-induced insulin resistance [33]. Furthermore, adipose tissue biopsies of obese individuals revealed that both subcutaneous adipose tissue and visceral adipose tissue exhibited γ-H2AX foci but that the visceral tissue exhibited 3-fold more foci [34]. Finally, in one study with obese children, peripheral blood lymphocytes were found to exhibit a greater than 5-fold increase in foci incidence in overweight children and 8-fold in obese children [35]. The normal cohort exhibited 0.0034±0.0006 foci per cell (fpc), overweight 0.019±0.0039 and obese 0.0274±0.0029 fpc. Micro-nuclear frequencies were also elevated in stimulated lymphocytes from the overweight and obese children compared to normal. These values for γ-H2AX foci incidence correspond to one per 294 cells in control individuals versus one per 37 in obese individuals. As with the mouse study, the elevated numbers of foci found were small, equivalent to approximately the number of foci observed 30 minutes after exposure to 2 mGy of ionizing radiation. However, the differences between the obese subjects and normal controls were substantially significant, attesting to the potential value of γ-H2AX foci measurements at low DSB levels.

In addition, there is a documented correlation between obesity and overall cancer incidence, as well as the incidence of other health issues. If increased levels of foci and DSB damage in lymphocytes and adipose tissue is indicative of increased damage levels elsewhere, these findings provide a possible molecular mechanism by which obesity may lead to increased cancer risk.

### Environmental genotoxicity and high-throughput assays

DSB detection by γ-H2AX foci has been used in studies of the effects of personal exposure to environmental agents such as air pollution, food toxins and industrial chemicals, all of which have the potential of large scale impacts on human populations as well as populations of other organisms. With the γ-H2AX assay researchers are able to monitor small amounts of genotoxicity *in vivo*, enabling them to evaluate the effects of environmental conditions on a population. As one practical example, a study with women in rural India exposed to high amounts of smoke from cooking with biomass fuel in poorly ventilated kitchens revealed a 4-fold elevation in the fraction of airway epithelial cells exhibiting γ-H2AX foci and 5-fold in peripheral blood lymphocytes compared to the ones using cleaner fuel liquefied petroleum gas [36]. These authors also found the elevation of other DNA damage markers. Such studies could provide evidence for governmental action to limit human exposure to certain environmental agents.

With the demonstrated utility of γ-H2AX foci measurements *in vivo*, new tools for high throughput γ-H2AX screenings are being developed. Audebert *et al*. reported the development of an *in vitro* genotoxicity assay system using γ-H2AX measurement to evaluate the effects of food poisons such as benzo(a)pyrene, fluoranthene, and 3-methylcholanthrene [37]. In addition to the automated high throughput RABIT system discussed above [17,18], one high-content screening assay [38] and two enzyme-linked immunosorbent assays (ELISA) that can accurately quantify γ-H2AX level, one for screening genotoxic molecules [39] and one for clinical trials [25] have been developed. These tools will greatly increase the capacity to identify genotoxic compounds, not only to humans, but also in other organisms. Since the H2AX phosphorylation site is highly conserved throughout eukaryotes, the γ-H2AX assay may find greatly expanded utility.

Finally, however, it should be kept in mind that while a γ-H2AX focus may indicate the presence of a DNA DSB in the vast majority of cases there are situations where one may be present without the other. The presence of γ-H2AX foci in the absence of DNA damage has been demonstrated by tethering single repair factors to chromatin [40]. Also, in normal senescent cell cultures, γ-H2AX may also be an indicator of mTOR-dependent senescent phenotype independent of DNA damage and of a classic DNA damage response [41]. In contrast, the presence of DSBs without γ-H2AX foci formation has been demonstrated in mouse kidney cells when bathed in high salt [42]. Break repair is hindered while the osmolality is high, but when it is returned to normal, γ-H2AX foci form and the breaks are repaired. Understanding these exceptional situations may help clarify the overall relationship between γ-H2AX foci and DSBs.

## CONCLUSIONS

The studies presented here demonstrate the wide range of genotoxic events *in vitro* and *in vivo* that are amenable to DNA damage measurements utilizing the γ-H2AX assay. In some cases there is substantial induction of γ-H2AX foci with exposure to high levels of ionizing radiation and cancer drugs, while in others the induction is of smaller magnitude, such as with diagnostic CT scans, environmental toxicity and chronic inflammation. γ-H2AX studies indicate a connection between DNA damage and chronic inflammation, but is the DNA damage observed during chronic inflammation just another indicator of a biological system that is not at optimum homeostasis, or does it have a causal roles on the long-term deleterious effects of chronic inflammation? Also, while the effects of large-scale DNA damage as indicated by strong γ-H2AX signals are readily apparent, it is not yet clear what a several-fold elevation of DNA damage markers including γ-H2AX foci mean to the organism either in the short-term or long-term. In the case of drug biodosimetry, the relationships of γ-H2AX responses of surrogate tissues relative to the γ-H2AX responses of tumors as well as the crucial biological responses of tumors, regression and disappearance, are beginning to be studied. These are all important questions that remain to be answered.

γ-H2AX studies are beginning to give useful input for decision making. Two examples discussed here are the comparison of various CT scanning protocols [10] and the choice of indenoisoquinolines for clinical evaluation [22]. While such studies to date have concentrated on human subjects, the almost universal conservation of DNA damage induced H2AX phosphorylation throughout eukaryotic evolution including plants suggests that future research may include many more subjects.
